# Supplementary figures and images for: DXA-derived hip shape is associated with hip fracture: a longitudinal study of 38 123 UK Biobank participants
Source: J Bone Miner Res. 2025 Nov 20;41(4):396–405. doi: 10.1093/jbmr/zjaf171 (PMC7618572; doi:10.1093/jbmr/zjaf171)

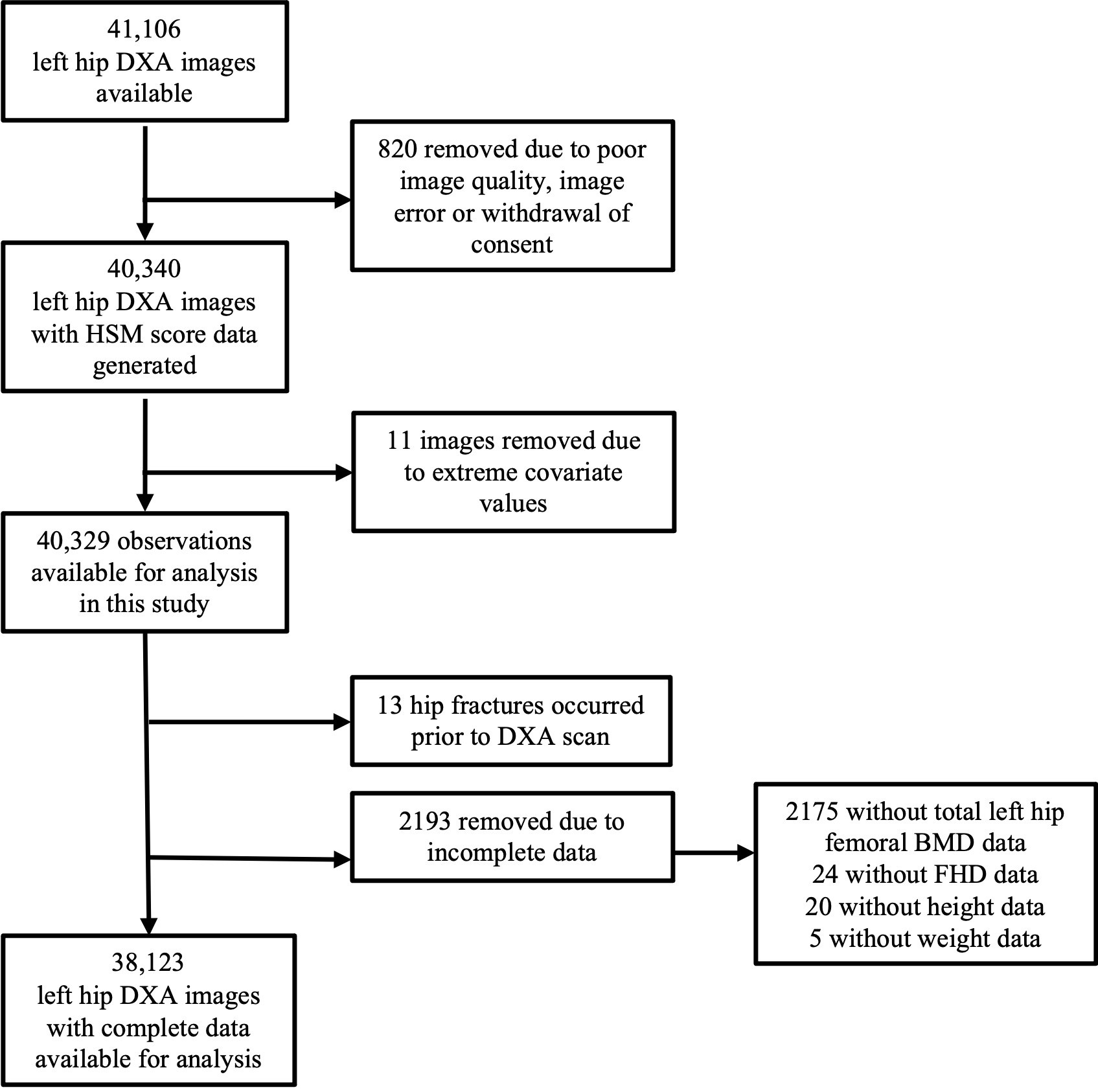

Supplement: Supplementary_figure_1_zjaf171 [file supplementary_figure_1_zjaf171.jpeg]

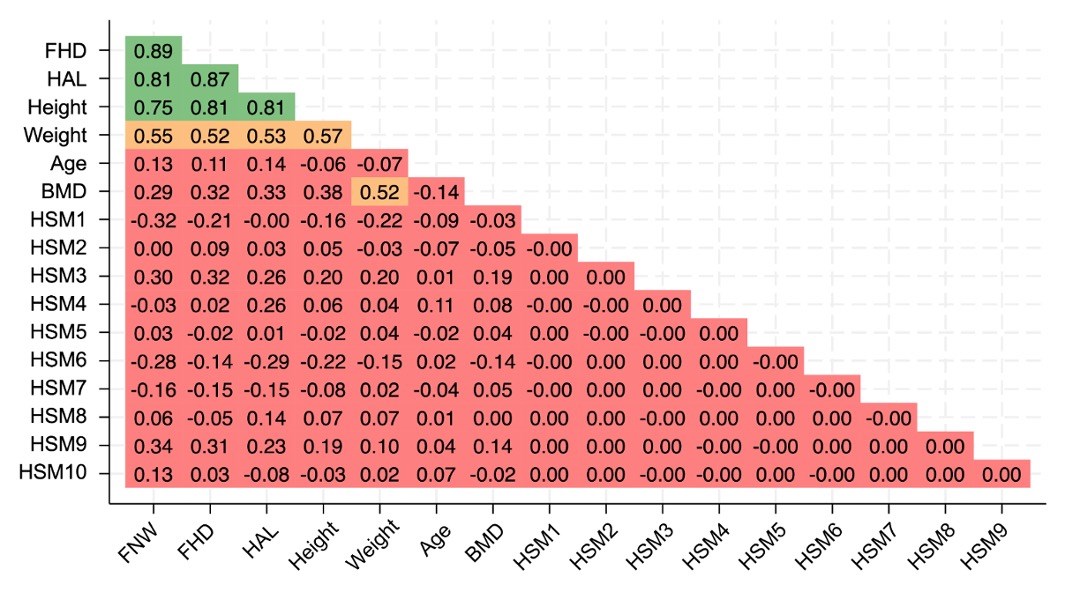

Supplement: Supplementary_figure_2_zjaf171 [file supplementary_figure_2_zjaf171.jpeg]

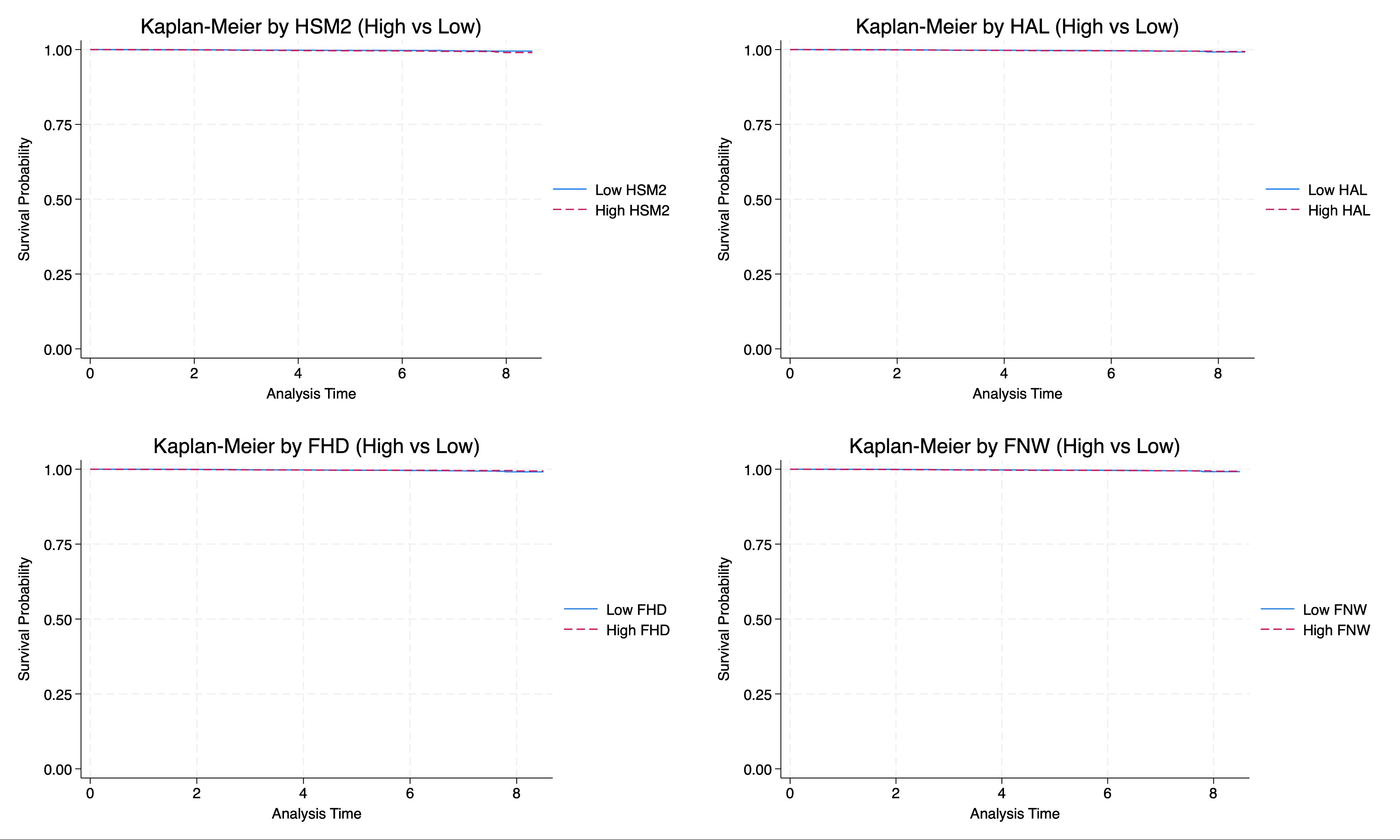

Supplement: Supplementary_Figure_3_zjaf171 [file supplementary_figure_3_zjaf171.jpeg]
