# Supplementary material for: DXA-derived hip shape is associated with hip fracture: a longitudinal study of 38 123 UK Biobank participants
Source: J Bone Miner Res. 2025 Nov 20;41(4):396–405. doi: 10.1093/jbmr/zjaf171 (PMC7618572; doi:10.1093/jbmr/zjaf171)
Supplement: SUPPLEMENTARY_TABLES_AND_FIGURES_zjaf171 [file supplementary_tables_and_figures_zjaf171.docx]

**SUPPLEMENTARY TABLES AND FIGURES**

**Supplementary Table 1: Distribution of hip fracture types**

|  | **ICD code** | **Fractures** |
| --- | --- | --- |
| Fracture of neck of femur | S72.0 | 103 |
| Pertrochanteric fracture | S72.1 | 26 |
| Subtrochanteric fracture | S72.2 | 3 |
| Stress fracture, not elsewhere classified (Pelvic region and thigh) | M84.359 | 1 |
| Pathological fracture | M84.459 | 0 |
| **Total** |  | **133** |

**Supplementary Table** **2: Ethnicity distribution and number of fractures in the study cohort**

| **Ethnicity** | **N (%)** | **Hip fractures** |
| --- | --- | --- |
| White | 36,861(96.7) | 131 |
| Asian | 424 (1.1) | 0 |
| Black | 250 (0.7) | 0 |
| Mixed | 174 (0.5) | 1 |
| Chinese | 115 (0.3) | 0 |
| Unknown | 299 (0.8) | 1 |
| **Total** | **38,123** | **133** |

**Supplementary Table 3: Correlation coefficient comparing DXA measures at baseline and follow up**

| **Measure** | **CCC** | **SE** | **Observations** | **LCI** | **UCI** | **P** | **CV (%)** |
| --- | --- | --- | --- | --- | --- | --- | --- |
| HSM1 | 0.85 | 0.02 | 354 | 0.82 | 0.88 | <0.001 | NA |
| HSM2 | 0.93 | 0.01 | 354 | 0.91 | 0.94 | <0.001 | NA |
| HSM3 | 0.82 | 0.02 | 354 | 0.79 | 0.85 | <0.001 | NA |
| HSM4 | 0.81 | 0.02 | 354 | 0.78 | 0.85 | <0.001 | NA |
| HSM5 | 0.51 | 0.04 | 354 | 0.43 | 0.58 | <0.001 | NA |
| HSM6 | 0.81 | 0.02 | 354 | 0.77 | 0.84 | <0.001 | NA |
| HSM7 | 0.70 | 0.03 | 354 | 0.65 | 0.76 | <0.001 | NA |
| HSM8 | 0.70 | 0.03 | 354 | 0.65 | 0.75 | <0.001 | NA |
| HSM9 | 0.72 | 0.02 | 354 | 0.68 | 0.77 | <0.001 | NA |
| HSM10 | 0.72 | 0.03 | 354 | 0.67 | 0.77 | <0.001 | NA |
| FNW | 0.99 | 0.00 | 354 | 0.99 | 0.99 | <0.001 | 1.70 |
| FHD | 0.99 | 0.00 | 353 | 0.99 | 0.99 | <0.001 | 1.27 |
| HAL | 0.99 | 0.00 | 354 | 0.99 | 0.99 | <0.001 | 1.03 |
| Total Left Femur BMD | 0.92 | 0.00 | 352 | 0.95 | 0.96 | <0.001 | 6.19 |
|  | **Mean (days)** | **Range (days)** |  |  |  |  |  |
| Time between scans | 835 | 735-979 |  |  |  |  |  |

CCC = concordance correlation coefficient, SE = standard error, LCI = lower confidence interval, UCI = upper confidence interval, CV = coefficient of variation, HSM = hip shape mode, FNW = femoral neck width, FDH = femoral head diameter , HAL = hip axis length , BMD = bone mineral density.

**Supplementary Table 4: Cox proportional hazard results for the association between each geometric measurement, ratios of the geometric measures, and hip fracture.**

|  | **Model 1** | | **Model 2** | | **Model 3** | | **Model 4** | |
| --- | --- | --- | --- | --- | --- | --- | --- | --- |
| **Exposure** | **HR [95% CI]** | **p-value** | **HR [95% CI]** | **p-value** | **HR [95% CI]** | **p-value** | **HR [95% CI]** | **p-value** |
| **Combined sex** |  |  |  |  |  |  |  |  |
| FNW (mm) | 1.15 [0.97-1.36] | 0.11 | 2.45 [1.80-3.33] | 1.17 × 10^-8^ | 1.77 [1.30-2.43] | 3.26 × 10^-4^ | 1.31 [0.88-1.96] | 0.19 |
| FHD (mm) | 1.12 [0.95-1.33] | 0.17 | 2.42 [1.76-3.32] | 5.20 × 10^-8^ | 1.89 [1.39-2.57] | 4.48 × 10^-5^ | 1.47 [0.96-2.25] | 0.07 |
| HAL (mm) | 1.08 [0.91-1.28] | 0.39 | 1.85 [1.34-2.55] | 1.93 × 10^-4^ | 1.61 [1.18-2.21] | 3.08 × 10^-3^ | 1.21 [0.84-1.74] | 0.31 |
| FNW/FHD | 1.12 [0.95-1.33] | 0.19 | 1.26 [1.04-1.52] | 0.02 | 1.09 [0.89-1.32] | 0.41 |  |  |
| FNW/HAL | 1.16 [0.98-1.37] | 0.08 | 1.33 [1.11-1.59] | 1.92 × 10^-3^ | 1.15 [0.96-1.38] | 0.14 |  |  |
| **Males** |  |  |  |  |  |  |  |  |
| FNW (mm) | 2.17 [1.44-3.25] | 1.99 × 10^-4^ | 2.13 [1.33-3.43] | 1.74 × 10^-3^ | 1.75 [1.08-2.82] | 0.02 | 1.17 [0.63-2.18] | 0.62 |
| FHD (mm) | 2.30 [1.54-3.44] | 4.50 × 10^-5^ | 2.36 [1.45-3.84] | 5.54 × 10^-4^ | 2.01 [1.28-3.14] | 2.26 × 10^-3^ | 1.61 [0.85-3.06] | 0.14 |
| HAL (mm) | 2.07 [1.37-3.11] | 4.84 × 10^-4^ | 2.01 [1.19-3.37] | 0.01 | 1.84 [1.11-3.04] | 0.02 | 1.39 [0.78-2.45] | 0.26 |
| FNW/FHD | 1.17 [0.85-1.61] | 0.33 | 1.12 [0.85-1.60] | 0.34 | 1.02 [0.74-1.42] | 0.89 |  |  |
| FNW/HAL | 1.21 [0.90-1.62] | 0.22 | 1.22 [0.90-1.64] | 0.20 | 1.10 [0.81-1.49] | 0.56 |  |  |
| **Females** |  |  |  |  |  |  |  |  |
| FNW (mm) | 2.88 [2.05-4.06] | 1.40 × 10^-9^ | 2.71 [1.80-4.08] | 1.63 × 10^-6^ | 1.70 [1.11-2.59] | 0.01 | 1.39 [0.82-2.35] | 0.22 |
| FHD (mm) | 2.43 [1.73-3.40] | 2.60 × 10^-7^ | 2.46 [1.62-3.74] | 2.36 × 10^-5^ | 1.70 [1.11-2.60] | 0.01 | 1.35 [0.77-2.36] | 0.29 |
| HAL (mm) | 2.05 [1.48-2.85] | 1.58 × 10^-5^ | 1.73 [1.15-2.62] | 0.01 | 1.40 [0.92-2.13] | 0.11 | 1.09 [0.67-1.75] | 0.73 |
| FNW/FHD | 1.42 [1.13-1.79] | 3.02 × 10^-3^ | 1.30 [1.03-1.65] | 0.03 | 1.10 [0.87-1.39] | 0.42 |  |  |
| FNW/HAL | 1.43 [1.14-1.79] | 1.87 × 10^-3^ | 1.41 [1.12-1.77] | 3.27 × 10^-3^ | 1.16 [0.92-1.47] | 0.20 |  |  |

Hazard ratios (HR) with 95% confidence intervals (CI) and p-values are shown for each geometric measure, and ratios of geometric measures and their association to hip fracture. HRs are reported per one standard deviation increase in each geometric measure. Model 1 = unadjusted; model 2 = adjusted for age, sex, height, and weight (no sex adjustment in sex-stratified analysis); model 3 = adjusted for model 2 plus bone mineral density; model 4 = adjusted for model 3 plus the remaining 2 geometric measures.

FNW = femoral neck width, FHD = femoral head diameter, HAL = hip axis length

**Supplementary Table 5: Cox proportional hazard results for the association between each hip shape mode and hip fracture in sex-stratified analysis**

|  | **Model 1** | | **Model 2** | | **Model 3** | | **Model 4** | |
| --- | --- | --- | --- | --- | --- | --- | --- | --- |
| **Exposure** | **HR [95% CI]** | **p-value** | **HR [95% CI]** | **p-value** | **HR [95% CI]** | **p-value** | **HR [95% CI]** | **p-value** |
| **Males** |  |  |  |  |  |  |  |  |
| Hip shape mode 1 | 1.12 [0.83-1.52] | 0.47 | 1.09 [0.80-1.47] | 0.59 | 1.28 [0.94-1.75] | 0.12 | 1.32 [0.89-1.97] | 0.17 |
| Hip shape mode 2 | 1.43 [1.06-1.93] | 0.02 | 1.39 [1.03-1.88] | 0.03 | 1.38 [1.02-1.86] | 0.04 | 1.34 [0.98-1.84] | 0.06 |
| Hip shape mode 3 | 1.20 [0.88-1.62] | 0.25 | 1.25 [0.92-1.69] | 0.16 | 1.27 [0.94-1.73] | 0.12 | 1.17 [0.86-1.60] | 0.33 |
| Hip shape mode 4 | 0.79 [0.59-1.07] | 0.13 | 0.77 [0.57-1.04] | 0.09 | 0.79 [0.58-1.07] | 0.13 | 0.72 [0.50-1.03] | 0.08 |
| Hip shape mode 5 | 0.84 [0.62-1.15] | 0.28 | 0.85 [0.63-1.16] | 0.32 | 0.92 [0.67-1.26] | 0.59 | 0.87 [0.63-1.20] | 0.40 |
| Hip shape mode 6 | 1.00 [0.73-1.36] | 1.00 | 0.99 [0.73-1.35] | 0.97 | 0.96 [0.70-1.31] | 0.79 | 0.94 [0.68-1.31] | 0.73 |
| Hip shape mode 7 | 1.10 [0.82-1.47] | 0.53 | 1.14 [0.85-1.52] | 0.39 | 1.20 [0.89-1.61] | 0.24 | 1.29 [0.96-1.74] | 0.09 |
| Hip shape mode 8 | 0.98 [0.73-1.32] | 0.89 | 0.98 [0.73-1.31] | 0.89 | 0.90 [0.67-1.21] | 0.48 | 0.96 [0.70-1.32] | 0.81 |
| Hip shape mode 9 | 0.72 [0.54-0.98] | 0.04 | 0.70 [0.52-0.95] | 0.02 | 0.73 [0.54-0.99] | 0.04 | 0.66 [0.48-0.89] | 0.01 |
| Hip shape mode 10 | 0.95 [0.72-1.26] | 0.73 | 0.94 [0.71-1.25] | 0.68 | 0.97 [0.74-1.28] | 0.84 | 0.91 [0.67-1.23] | 0.53 |
| **Females** |  |  |  |  |  |  |  |  |
| Hip shape mode 1 | 0.83 [0.67-1.03] | 0.09 | 0.87 [0.70-1.09] | 0.22 | 1.02 [0.82-1.28] | 0.83 | 1.04 [0.80-1.36] | 0.76 |
| Hip shape mode 2 | 1.27 [1.04-1.56] | 0.02 | 1.37 [1.11-1.68] | 2.79 × 10^-3^ | 1.26 [1.03-1.54] | 0.02 | 1.27 [1.03-1.57] | 0.02 |
| Hip shape mode 3 | 1.05 [0.84-1.31] | 0.68 | 1.07 [0.86-1.34] | 0.54 | 1.13 [0.90-1.40] | 0.29 | 1.08 [0.86-1.35] | 0.52 |
| Hip shape mode 4 | 0.97 [0.78-1.19] | 0.75 | 0.87 [0.71-1.08] | 0.21 | 0.94 [0.76-1.15] | 0.54 | 0.96 [0.75-1.23] | 0.76 |
| Hip shape mode 5 | 1.01 [0.83-1.24] | 0.89 | 1.05 [0.86-1.27] | 0.65 | 1.05 [0.86-1.29] | 0.60 | 1.03 [0.84-1.26] | 0.78 |
| Hip shape mode 6 | 1.03 [0.84-1.27] | 0.78 | 1.01 [0.82-1.24] | 0.96 | 0.96 [0.78-1.18] | 0.68 | 0.96 [0.76-1.22] | 0.75 |
| Hip shape mode 7 | 0.87 [0.71-1.07] | 0.18 | 0.94 [0.77-1.15] | 0.56 | 0.99 [0.81-1.22] | 0.96 | 1.04 [0.84-1.27] | 0.73 |
| Hip shape mode 8 | 1.05 [0.85-1.29] | 0.68 | 1.04 [0.85-1.28] | 0.70 | 0.98 [0.80-1.21] | 0.87 | 1.03 [0.82-1.29] | 0.82 |
| Hip shape mode 9 | 1.14 [0.91-1.41] | 0.25 | 1.15 [0.92-1.43] | 0.22 | 1.15 [0.92-1.44] | 0.21 | 1.07 [0.85-1.35] | 0.56 |
| Hip shape mode 10 | 1.01 [0.81-1.26] | 0.92 | 0.97 [0.78-1.21] | 0.80 | 0.96 [0.77-1.19] | 0.68 | 0.91 [0.72-1.14] | 0.40 |

Hazard ratios (HR) with 95% confidence intervals (CI) and p-values are shown for each hip shape mode and their association to hip fracture. HRs are reported per one standard deviation increase in each hip shape mode. Model 1 = unadjusted; model 2 = adjusted for age, height, and weight; model 3 = adjusted for model 2 plus bone mineral density; model 4 = adjusted for model 3 plus the geometric measures.

**Supplementary Table 6:** **Cox proportional hazard results for the associations between bone mineral density and hip fracture.**

|  | **Model 1** |  | **Model 2** |  |
| --- | --- | --- | --- | --- |
| **Exposure** | **HR [95% CI]** | **p-value** | **HR [95% CI]** | **p-value** |
| Combined sex | 0.37 [0.32-0.42] | 1.76 × 10^-43^ | 0.36 [0.30-0.43] | 2.86 × 10^-28^ |
| Male | 0.38 [0.28-0.51] | 2.70 × 10^-10^ | 0.38 [0.28-0.53] | 4.74 × 10^-9^ |
| Female | 0.37 [0.31-0.44] | 9.79 × 10^-32^ | 0.34 [0.27-0.43] | 2.71 × 10^-20^ |

Hazard ratios (HR) with 95% confidence intervals (CI) and p-values are shown for the association between bone mineral density and hip fracture in combined-sex and sex-stratified analysis. HRs are reported per one standard deviation increase in bone mineral density. Model 1 = unadjusted; model 2 = adjusted for age, sex, height, and weight (no sex adjustment in sex-stratified analysis).


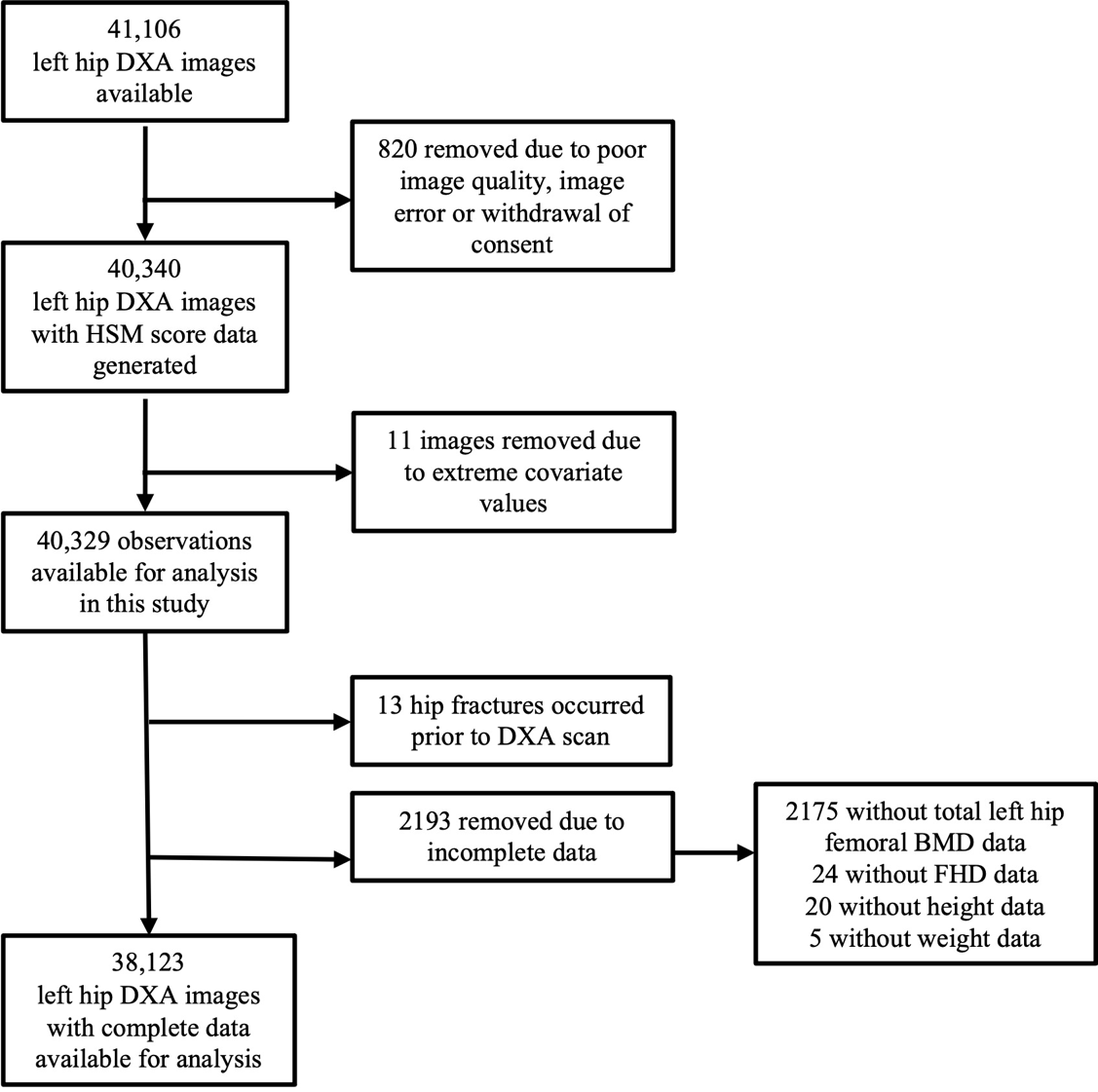


**Supplementary Figure 1: Flow diagram showing inclusion and exclusion criteria of the left hip DXA images available for analysis from UK Biobank.**

DXA = dual-energy X-ray absorptiometry, HSM = hip shape mode, BMD = bone mineral density, FHD = femoral head diameter.

**
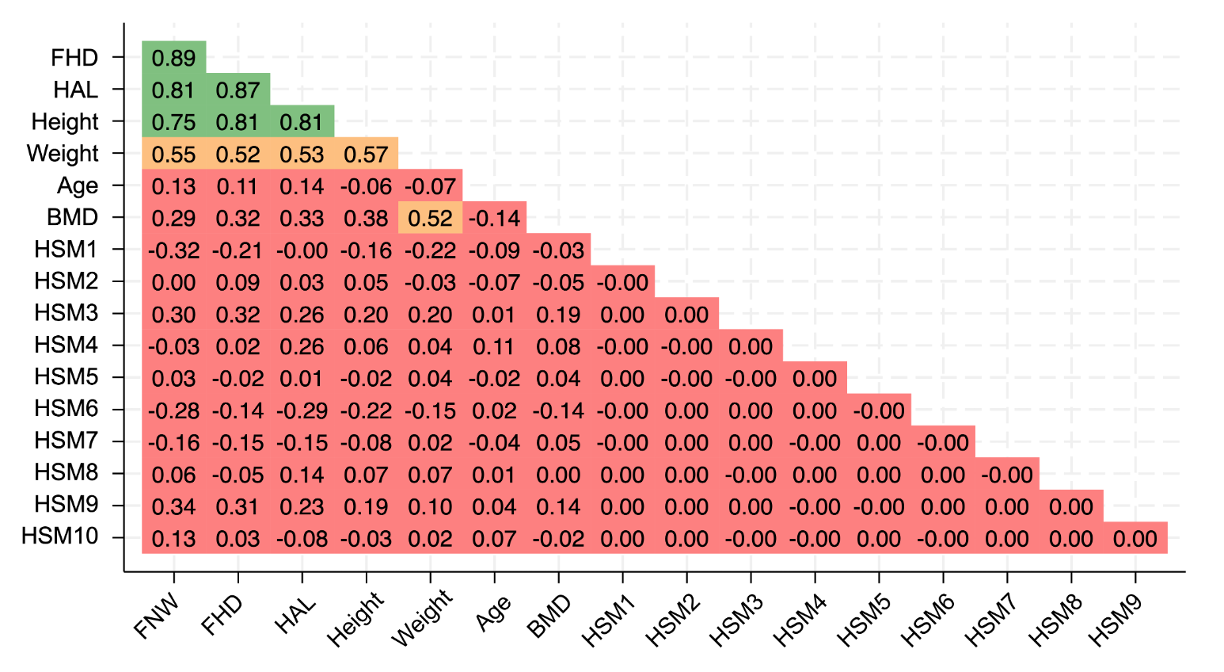
**

**Supplementary Figure 2: Pearson’s correlation matrix (r) showing the correlation between each HSM, GM (FHD, HAL, FNW), height, weight, age and BMD within the cohort.** Green shows a strong correlation (r ≥0.7-1), orange shows a moderate correlation (r ≥0.5-<0.7), red shows a weak correlation (r <0.5).

FHD = femoral head diameter, HAL = hip axis length, FNW = femoral neck width, BMD = bone mineral density, HSM = hip shape mode, GM = geometric measure.

**
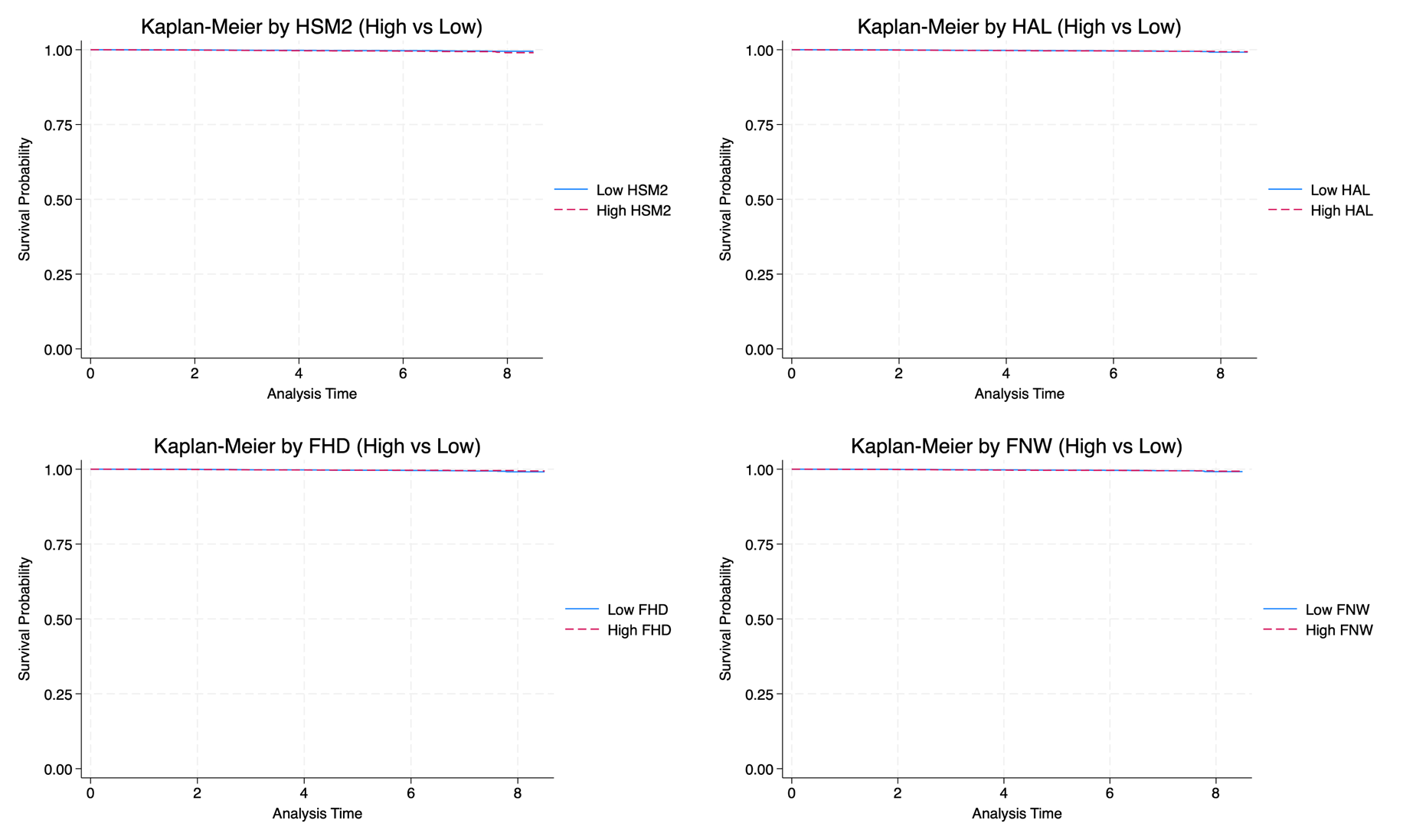
**

**Supplementary Figure 3: Kaplan-Meier curves showing survival analysis over time for HSM2 and each GM (HAL, FHD, FNW).** Curves were stratified by the median value of each variable, with high values defined as above the median and low values defined as below the median for HSM2 and each GM. Due to the small number of fractures, survival curves show minimal divergence between groups.

HSM = hip shape mode, GM = geometric measure, HAL = hip axis length, FHD = femoral head diameter, FNW = femoral neck width.
